# Measurements of Objective Cyclotorsion in a Population of Healthy Children

**DOI:** 10.1155/joph/6982201

**Published:** 2024-12-19

**Authors:** Hsu-Ying Lin, Wei-Chi Wu, Ming-Hui Sun, Jui-Yen Lin, Ping-Hsuan Huang, Chun-Hsiu Liu

**Affiliations:** ^1^Department of Ophthalmology, Chang Gung Memorial Hospital, Keelung, Taiwan; ^2^Department of Ophthalmology, Chang Gung Memorial Hospital, Linkou Medical Center, Taoyuan, Taiwan; ^3^College of Medicine, Chang Gung University, Taoyuan, Taiwan; ^4^Department of Ophthalmology, New Taipei City Tucheng Hospital, New Taipei City, Taiwan; ^5^Center of Statistics and Big Data, Chang Gung Memorial Hospital, Linkou Medical Center, Taoyuan, Taiwan

**Keywords:** Bland–Altman plots, cyclodeviation, cyclotropia, ocular torsion angle, optical coherence tomography, prematurity

## Abstract

**Introduction:** This study aimed to compare ocular torsion measurements to investigate normative objective cyclotorsion values in a population of healthy, full-term and preterm children.

**Materials and Methods:** The participants enrolled in this study had an age range of 3–12 years and were divided into two groups, full-term (gestational age (GA) > 37 weeks) and preterm without retinopathy of prematurity (ROP) (GA ≤ 37 weeks). The disc-center-fovea angle (DFA) was used to evaluate ocular torsion using two different imaging modalities: optical coherence tomography (OCT) with a 55-degree field of view (FV) and conventional fundus photography (CFP) with a 45-degree FV. The values measured from both right and left eyes were combined to obtain a single value to compensate for the effect of head tilt during measurement.

**Results:** A total of 86 full-term and 145 preterm children were enrolled in this study. The DFAs measured using OCT and CFP were −11.57° ± 5.27° and −12.07° ± 5.66° in the full-term group and −10.64° ± 5.40° and −11.25° ± 4.80° in the preterm group, respectively. There were no significant differences between the results obtained from OCT and CFP in the two groups (*p*=0.109 and *p*=0.512, respectively). There was a strong correlation between OCT and CFP in all patients, with a Pearson's correlation coefficient of 0.74 and an intraclass correlation coefficient (ICC) of 0.74 (both *p* < 0.001). Multivariate regression analysis showed that the average axial length (AXL) was associated with DFA.

**Conclusions:** This study found a significant correlation between DFA measured using OCT and CFP, making either measurement modality feasible in pediatric populations. There was no significant difference in the DFA between full-term and preterm children. AXL demonstrated an association with the DFA.

## 1. Introduction

Accurate cyclodeviation measurements are essential for the effective diagnosis and treatment of strabismus [1]. The assessment of torsional angles includes subjective measurements, such as the synoptophore and double Maddox rod tests, and objective measurements, such as fundus photography. However, subjective techniques that rely on patient cooperation can be challenging to implement, especially in young children who may face difficulties complying with the required examination procedures. Consequently, objective measurement methods have become highly valuable for obtaining reliable and reproducible results in pediatric patients.

Numerous studies have compared variations in ocular torsion measurements obtained using various fundus photography methods [2, 3]. Although optical coherence tomography (OCT)–based techniques have been reported for measuring the ocular torsion angle in adults, there is currently no research on their application in children. Additionally, proper head alignment is essential during fundus image–based measurements [4]. Novel analytical techniques are required to address the potential errors caused by subtle head tilts in young subjects, which can adversely affect the precision of cyclodeviation measurements. These methods can yield more accurate and reliable cyclodeviation measurements in children. Considering that a small head tilt angle in healthy individuals induces a corresponding and compensatory rotation of both the right and left eyes [5], we propose a novel method for calculating the torsional angle by summing the torsional angles of both eyes.

Previous studies have reported that preterm birth may be related to several ophthalmic conditions [6, 7]; however, there is limited research on the potential variations in cyclodeviation between healthy full-term and preterm children. Therefore, to address these issues, the current study aimed to compare ocular torsion measurements obtained using different fundus photography techniques in full-term and preterm children.

## 2. Materials and Methods

This retrospective observational study was approved by the Institutional Review Board of Chang Gung Memorial Hospital in Taiwan (202201840B0). All the procedures complied with the principles of the Declaration of Helsinki. The parents of each child provided written informed consent.

### 2.1. Study Design and Patient Grouping

Patients were divided into the following two groups based on age: full-term group, which included children who did not experience preterm birth (gestational age (GA) ≥ 37 weeks), and preterm group, which included children who were born prematurely (GA ≤ 37 weeks) and had no prior history of retinopathy of prematurity (ROP).

### 2.2. Ophthalmic Examinations

An autorefractokeratometer (ARK-1; Nidek, Aichi, Japan) was used to perform cycloplegic refraction. An optical biometer (IOL Master; Carl Zeiss, Jena, Germany) was used to measure the axial length (AXL) and anterior chamber depth (ACD). A single experienced ophthalmologist (W.C.W.) performed an indirect fundus examination of each patient, which was included as part of a thorough ophthalmic evaluation. Study eyes or individuals with any of the following conditions were excluded: (1) insufficient image quality or poor photograph clarity for determining the location of the fovea; (2) any optic disc pathological feature, such as optic disc coloboma or hypoplasia; (3) any macula diseases that affect the fovea, such as macular edema or macular dragging/scarring; (4) any type of strabismus; (5) previous ocular surgery, such as blowout fracture, retinal detachment, or strabismus surgery; or (6) limitation of eye movement in duction/version testing.

### 2.3. Fundus Torsion Measurements

We used two different mydriatic fundus photography techniques to objectively measure ocular torsion in both eyes: OCT with a 55-degree field of view (FV) (Spectralis, Heidelberg Engineering, Heidelberg, Germany) and conventional fundus photography (CFP) with a 45-degree FV (nonmyd 8s, KOWA, Tokyo, Japan).

Each eye was examined separately in the primary position. The patients were instructed to gaze at an internal fixation target for measurements that required internal fixation. Patients were instructed to maintain the same position throughout both tests using chin and forehead supports to prevent head tilting. Each eye was examined separately, while maintaining the same head position. Both tests were performed on the same day. Throughout the measurements, the patients were instructed to keep both eyes open. Data from uncooperative patients were excluded from analysis. A single sharp photograph was used to compute the disc-center-fovea angle (DFA).

**Figure 1:**
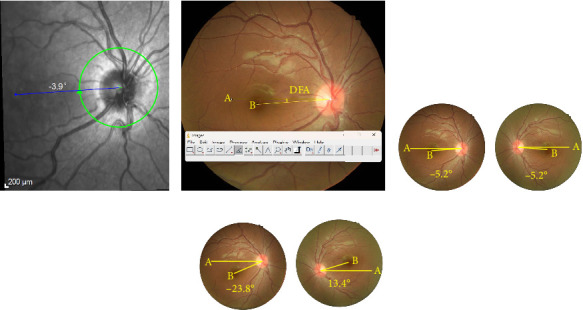
Measurement of the disc-center-fovea angle (DFA). (a) Measuring the DFA using the spectral domain OCT system; the built-in software automatically measured DFA. The picture illustrates an ocular torsion angle of −3.9°. (b) Measuring the DFA using the ImageJ software. The DFA is determined by drawing a horizontal line “A” from the center of the optic disc and another line “B” connecting the fovea to the center of the optic disc. (c, d) Angle differences caused by the tilting of the head in the same patient. The combined value of both eyes remains the same (−10.4°), but the individual eye values change due to the head tilt. (c) DFA without head tilt. (d) DFA with head tilted.

The automatic DFA was measured on the image obtained with the spectral domain OCT system using built-in software (Version 1.10.2.0) (Figure 1(a)). A single-blinded ophthalmologist (H.Y.L.) carefully examined the scanned images. Any errors discovered during the automatic DFA measurement were manually adjusted. Data from both the right and left eyes were combined to eliminate head-tilt–related errors for subsequent analysis.

With the aid of standard image analysis software (Version 1.53t, ImageJ; developed by Wayne Rasband, National Institutes of Health, Bethesda, MD, USA) (Figure 1(b)), the torsional angle was measured between a horizontal line passing through the center of the optic disc and a line joining the center to the fovea. The center of the optic disc was identified as described previously [8]. The height and width of the optic disc were manually measured and used to fit a rectangle. The intersection of the two diagonal lines served as the center. The degree of ocular torsion was measured using this method. The intorsional degree was defined as a positive value, whereas the extorsional degree was defined as a negative value. One skilled examiner (H.Y.L.), who was blinded to patient information, examined each image. Data from both eyes were combined to eliminate head-tilt–related errors for subsequent analysis (Figures 1(c) and 1(d)).

### 2.4. Statistical Analysis

Means, medians, and ranges were calculated for continuous variables, whereas the numbers of values and percentages in each category were determined for discrete variables. Differences between groups were evaluated using the Wilcoxon signed-rank test for continuous variables and the chi-squared test for categorical variables. To compare the disparities in ocular torsion angle measurements between OCT and the fundus camera, a Bland–Altman plot was used. Intraclass correlation coefficients (ICCs) were calculated to evaluate the agreement between measures. ICC > 0.7 was considered significant, indicating good agreement between the two methods.

Simple linear regression was used to determine potential associations between age, sex, refractive error (spherical equivalent refraction (SE)), AXL, and best-corrected visual acuity (BCVA). Multiple linear regression analysis was conducted to investigate the association of the factors identified during the simple regression analysis with the ocular torsion angle measurements. Statistical analyses were conducted using the SAS software (Version 9.4; SAS Institute, Cary, NC, USA). All the analyses performed reported *p* values, and statistical significance was defined as *p* < 0.05.

## 3. Results

A total of 232 patients were enrolled in this study.  Table 1 shows the birth and ocular characteristics of the study groups. The mean of GA and birth weight were significantly greater in the full-term group (38.73 ± 1.55 weeks and 3166.22 ± 429.20 g) than in the preterm group (38.73 ± 1.55 weeks and 1670.01 ± 574.95 g) (both *p* < 0.001). At the time of enrollment, the mean age of the patients in the full-term group (9.08 ± 2.04 years) was significantly higher than that in the preterm group (7.4 ± 2.39 years) (*p* < 0.001). The mean AXL was significantly greater while the mean SE and BCVA (log MA) were significantly lower in the full-term group (23.81 ± 1.01 mm, −0.42 ± 1.81, and 0 ± 0.03 and 22.93 ± 1.15 mm, 0.07 ± 1.76, and 0.01 ± 0.05 for the full-term and preterm groups, respectively; *p* < 0.001, *p*=0.008, and *p*=0.036).

### 3.1. Measurements of the Ocular Torsion Angle

**Figure 2:**
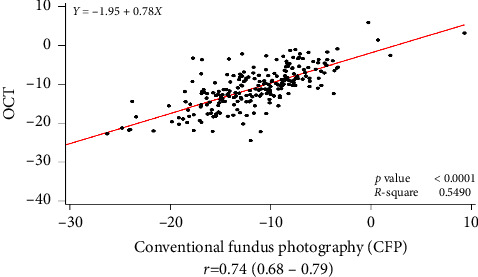
Pearson's correlation of the disc-center-fovea angle (DFA) measured by conventional fundus photography (CFP) and optical coherence tomography (OCT).

Table 2 compares the measurements of ocular torsion angle between the two study groups. DFA measured using OCT and CFP showed no significant differences between the groups (*p*=0.109 and *p*=0.512, respectively). OCT and CFP provided equivalent measurements of ocular torsion angle. OCT and CFP measurements of the ocular torsion angle showed sufficient correlation when analyzed with Pearson's correlation (*p* < 0.001, *R* = 0.74 in all patients) (Figure 2).

### 3.2. Bland–Altman Plots and Intraclass Correlation

**Figure 3:**
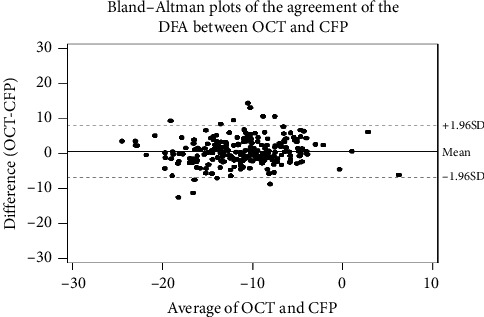
Bland–Altman plots representing the agreement of the disc-center-fovea angle (DFA) measured by conventional fundus photography (CFP) and optical coherence tomography (OCT).

The agreement of ocular torsion angle measurements between OCT and CFP was verified using Bland–Altman plots (Figure 3). In all the patients, the mean difference between OCT and CFP was 0.62°. The Bland–Altman plots used for comparisons revealed good agreement between OCT and CFP. The ICC for consistency between the two methods was 0.74.

### 3.3. Univariate and Multivariate Predictors

In the univariate linear regression, parameters including birth weight and mean SE were independently associated with DFA, as evaluated by CFP. A condition index greater than 30 indicated the presence of high collinearity. There was high collinearity between the parameters of birth weight and GA (index value 55.97) and birth weight and mean AXL (index value 134.14), as well as between mean SE and mean AL (index value 103.16). These pairs exhibited relatively high correlation; therefore, one variable from each pair was selected for inclusion. In multivariate linear regression with different covariates, only AXL was an important predictor of DFA measured with CFP (Table 3).

## 4. Discussion

In this study, the ocular torsion angle in children was measured using OCT and CFP, and the torsion angles between premature and non-premature children were compared. To the best of our knowledge, our study is the first to focus on comparing the ocular alignment status of children in these two cohorts, while adapting a novel approach to resolve the issue of head tilting during torsion measurement. We found no significant differences in DFA measurements between premature and non-premature children. DFA measured using OCT and CFP showed a significant correlation.

Numerous subjective techniques have been used to measure cyclotorsion, such as the double Maddox rod test and the synoptophore [9–11]. However, as it requires accurate subjective reactions, its use in young children remains challenging. In children, objective methods using structural measurements are generally easier and more feasible than subjective methods. In the past, photography was the traditional method used for objective methods of measuring cyclotorsion. In normal individuals, it is widely accepted that a horizontal line drawn from the fovea intersects the lower one-third diameter of the optic disc [12, 13]. However, in this traditional method, grading needs to be performed with a CFP, and a marking template must be placed over the CFP, which makes the method cumbersome [1]. Fundus photography employing DFA is currently considered the gold standard for the objective measurement of ocular torsion [14, 15]. In recent years, modified methods using OCT have been introduced to measure DFA [2, 3, 16–18].

However, all of the above examinations have one prerequisite: The head must be straight and not tilted. Therefore, cooperative issues still arise when working with children, even with objective approaches that use structural measures. The gold standard approach often requires the patient's head to be in the correct position without tilting, even with objective approaches that use structural measures. The ocular counter-rolling response, which occurs in the direction opposite to the head tilt, is elicited by lateral head tilting [19]. The effects of variations in head position on retinal imaging have been discussed in a few studies, along with the possibility that these changes can result in measurement errors [4, 5, 20]. A previous study demonstrated a significant association between a small degree of head tilt and rotational shifts in fundus photographs of both eyes, and the values did not change depending on whether the head tilt was to the right or left for both eyes [5]. Therefore, as the rotational angles of both eyes are relative to each other, we believe that summing the angles of both eyes can provide a comparative measure of their relative positions. This approach can help mitigate the issue of head tilt and provide a more comprehensive understanding of the eye rotation in relation to each other. This may be a useful strategy to consider when analyzing data and interpreting results.

OCT detects retinal information using high-contrast infrared images, making it possible to assess patients who are challenging to quantify with fundus photography because of small pupils or opacities of the optic media [3]. The central position of the fovea cannot be properly determined when measuring the DFA with CFP, and manual measurements may lead to bias. OCT can be used to perform cross-sectional macular scans to verify the correct location of the fovea. Consequently, it offers more accurate information than CFP [18]. OCT is particularly practical because it provides automated angle measurements [16, 21].

Our research revealed a good agreement between OCT and CFP (ICC = 0.74). Previous literature has shown no discernible difference between OCT and CFP because the DFA obtained using OCT is closely associated with that measured using CFP [2, 16]. Because of the abovementioned advantages of measuring DFA with OCT and the high correlation with DFA measured using CFP, it is feasible that in the future, DFA measurements using OCT alone would be sufficient.

The DFAs measured in our study by summing the values of both eyes were compatible with the measurements reported in the previous literature. Children aged 3–11 years had a normal ocular torsion angle of 6.89° ± 4.41° per eye when measured with non-mydriatic CFP [22]. In accordance with the previously reported values in children [22–24], the DFAs summing up both eyes in our study using OCT and CFP were −11.57° ± 5.27° and −12.07° ± 5.66° in the full-term group and −10.64° ± 5.40° and −11.25° ± 4.80° in the preterm group, respectively. Previous literature has shown that the angles were generally similar between adults and children [23]. Previous studies have reported the normal angle in adults as −5.6° ± 3.3° [13] and −7.25° ± 2.57 per eye [12] when measured using fundus photography, where the fovea is located at the lower one-third portion of the optic disc [12]. Kang et al. [2] reported that the DFAs for the right and left eyes measured using CFP with an 45-degree FV and OCT with a 55-degree FV in healthy adults were 5.39° ± 2.65° and 5.71° ± 3.16° and 5.27° ± 2.67° and 5.72° ± 3.20°, respectively. Based on these results, we can infer that there are no significant differences in the rotational angles of the eyes between children and adults. In this study, age was not found to be associated with the DFA in multivariate linear regression, which further supports this conclusion.

The DFA was not found to be associated with prematurity but was found to be related to AXL. We observed that the full-term group had a higher age compared to the preterm group. We believe that this age difference contributed to the longer AXL and the lower SE and logMAR values observed in the full-term group. These findings align with the trends observed in the literature regarding age and normal development in children [25–27]. AXL was found to be associated with the DFA in multiple linear regression. The relationship between the disc-fovea distance, AXL, and DFA has been discussed in the literature [28, 29]. Our results reported similar findings, where longer AXL is associated with smaller angle values and more “intorsional” angle. It was hypothesized that ametropia caused by AXL variations could lead to variable angular separations between the macula and optic disc as a result of magnification [30]. However, some authors claimed that the DFA and refraction only have a weak relationship [13, 17, 24]. Nevertheless, these authors did not analyze whether the ametropia cases originated from AXL differences [13, 17, 24]. Lengwiler et al. [17] excluded more extreme cases of ametropia from their study. The aforementioned factors are the driving forces behind various conclusions. Additional research is required to elucidate the correlation between these factors and the DFAs.

This study has several limitations. The retrospective design of this study may have resulted in bias. Second, the potential differences with or without dilation should be considered. High-quality imaging is important in uncooperative children, especially when considering the impact of dilation. To improve the image quality of CFP in this study, we employed mydriatic images. Nevertheless, concerns have been raised regarding the potential impact of dilated examinations on fixation. It has been suggested that a blurred image resulting from dilation could potentially disrupt the patient's ability to maintain stable fixation [2]. However, another study showed that regardless of whether the patients underwent fixation or eye tracking, the angles assessed were comparable [17]. As a result, we believe that the existence of dilatation and its potential impact on fixation in the current study had little to no meaningful impact on our conclusions. Third, head tilt only produces an equal amount and corresponding cyclotorsion in both eyes in subjects with a normal ocular counter-rolling reflex and no ocular motility abnormalities [19]. Therefore, our calculation method may not be applicable to patients with ophthalmological or vestibular abnormalities. Nevertheless, because we studied these angles in healthy children, our conclusions were not affected. Fourth, we did not have any AXL outliers in this study; therefore, the interpretation of AXL values beyond a certain range is uncertain. Finally, we did not conduct longitudinal tracking, which should have had a minimal impact because age was not found to be related to the DFA.

## 5. Conclusion

Our study demonstrated a significant correlation between DFA measured using OCT and CFP. In addition, we offered normative values for cyclotorsion that can probably be used by clinicians as reference values in clinical practice. However, because AXL is related to DFA, caution may be necessary when dealing with extreme AXL cases, and further surveys are required to better understand the concept of AXL outliers.

## Figures and Tables

**Table 1:** Patient birth and ocular characteristics.

	Full-term group (*n* = 86)	Preterm group (*n* = 146)	*p* value⁣^∗^
Age at examination (y)	9.08 ± 2.04	7.40 ± 2.39	< 0.001
Male sex	53 (61.6%)	75 (51.4%)	0.129
Gestational age (wk)	38.73 ± 1.55	32.16 ± 2.81	< 0.001
Birth weight (g)	3166.22 ± 429.20	1670.01 ± 574.95	< 0.001
Apgar score at 1 min	8.81 ± 0.48	7.37 ± 1.60	< 0.001
Apgar score at 5 min	9.75 ± 0.71	8.88 ± 1.22	< 0.001
Mean^†^ of best-corrected visual acuity in logMAR	0.00 ± 0.03	0.01 ± 0.05	0.036
SD^†^ of best-corrected visual acuity in logMAR	0.00 ± 0.02	0.01 ± 0.03	0.043
Mean^†^ of spherical equivalent (D)	−0.42 ± 1.81	0.07 ± 1.76	0.008
SD^†^ of spherical equivalent (D)	0.36 ± 0.43	0.30 ± 0.33	0.370
Mean^†^ of axial length (mm)	23.81 ± 1.01	22.93 ± 1.15	< 0.001
SD^†^ of axial length (mm)	0.15 ± 0.19	0.11 ± 0.14	0.098

*Note:* Data are presented as mean ± standard deviation or count (percent).

Abbreviation: SD, standard deviation.

⁣^∗^Wilcoxon rank-sum test or chi-square test.

^†^Mean and standard deviation of bilateral ocular measurements.

**Table 2:** Measurements of the ocular torsion angle.

	Full-term group (*n* = 86)	Preterm group (*n* = 146)	*p* value⁣^∗^
OCT	−11.57 ± 5.27	−10.64 ± 5.40	0.109
CFP	−12.07 ± 5.66	−11.25 ± 4.80	0.512

*Note:* Data are presented as mean ± standard deviation. OCT: DFA measured by OCT; CFP: DFA measured by CFP.

⁣^∗^Wilcoxon rank-sum test.

**Table 3:** Univariate and multivariate linear regression analysis: role of parameters on DFA measured by CFP in healthy children.

Covariate	Univariate				Multivariate			
	β	SE	95% CI	*p* value	β	SE	95% CI	*p* value
Age at examination (y)	0.011	0.141	−0.2669–0.2888	0.9381				
Male sex	−0.9701	0.6764	−2.3029–0.3626	0.1529	−0.8164	0.7485	−2.292–0.6592	0.2766
Gestational age (wk)	−0.1137	0.0845	−0.2801–0.0528	0.1797	−0.2012	0.1147	−0.4274–0.025	0.081
Birth weight (g)	−0.0008	0.0004	−0.0015–0	0.0408				
Apgar score at 1 min	−0.3154	0.2338	−0.7761–0.1454	0.1787	−0.2294	0.2941	−0.8093–0.3505	0.4364
Apgar score at 5 min	−0.3112	0.3036	−0.9094–0.2871	0.3065				
Mean^†^ of best-corrected visual acuity in logMAR	5.6937	7.9251	−9.9224–21.3098	0.4732				
SD^†^ of best-corrected visual acuity in logMAR	1.7325	15.0573	−27.9388–31.4038	0.9085				
Mean^†^ of spherical equivalent (D)	−0.6222	0.1889	−0.9945–0.2499	0.0012				
SD^†^ of spherical equivalent (D)	−0.7551	0.9323	−2.5925–1.0824	0.4189				
Mean^†^ of axial length (mm)	0.5129	0.3012	−0.0807–1.1065	0.09	0.7447	0.3425	0.0695–1.4198	0.0308
SD^†^ of axial length (mm)	−1.6551	2.2028	−5.9969–2.6867	0.4532				

^†^Mean and standard deviation of bilateral ocular measurements.

## Data Availability

The datasets used and/or analyzed during the current study are available from the corresponding author on reasonable request.

## Nomenclature

ROP: Retinopathy of prematurity
GA: Gestational age
DFA: Disc-center-fovea angle
OCT: Optical coherence tomography
FV: Field of view
CFP: Conventional fundus photography
AXL: Axial length
SE: Spherical equivalent refraction
BCVA: Best-corrected visual acuity
ACD: Anterior chamber depth
